# Valley-controlled photoswitching of metal–insulator nanotextures

**DOI:** 10.1038/s41567-025-02899-5

**Published:** 2025-05-21

**Authors:** Hannes Böckmann, Jan Gerrit Horstmann, Felix Kurtz, Manuel Buriks, Karun Gadge, Salvatore R. Manmana, Stefan Wippermann, Claus Ropers

**Affiliations:** 1 Max Planck Institute for Multidisciplinary Sciences, Göttingen, Germany; 2 IV. Physical Institute, Solids and Nanostructures, University of Göttingen, Göttingen, Germany; 3 Department of Materials, ETH Zurich, Zürich, Switzerland; 4 Institute for Theoretical Physics, University of Göttingen, Göttingen, Germany; 5 Department of Physics, Philipps-Universität Marburg, Marburg, Germany

**Keywords:** Phase transitions and critical phenomena, Surfaces, interfaces and thin films, Nanowires, Electronic properties and materials

## Abstract

Spatial heterogeneity and phase competition are hallmarks of strongly correlated materials, influencing phenomena such as colossal magnetoresistance and high-temperature superconductivity. Active control over phase textures further promises tunable functionality at the nanoscale. Although light-induced switching of a correlated insulator to a metallic state is well established, optical excitation generally lacks the specificity to select subwavelength domains and determine final textures. Here we drive the domain-specific quench of a textured Peierls insulator using valley-selective photodoping. Polarized excitation exploits the anisotropy of quasi-one-dimensional states at the charge-density-wave gap to initiate an insulator–metal transition with minimal electronic heating. We find that averting dissipation facilitates domain-specific carrier confinement, control over nanotextured phases and reduction in thermal relaxation from the metastable metallic state. This valley-selective photoexcitation approach will enable the activation of electronic phase separation beyond thermodynamic limitations, facilitating optically controlled hidden states, engineered heterostructures and polarization-sensitive percolation networks.

## Abstract (web summary)

Optical excitation is a way to control phase transitions in materials, but it lacks spatial specificity. Now, valley-selective photodoping in a Peierls insulator is demonstrated as a method to optically engineer phase textures with minimal heating.

## Main

**Fig. 1:**
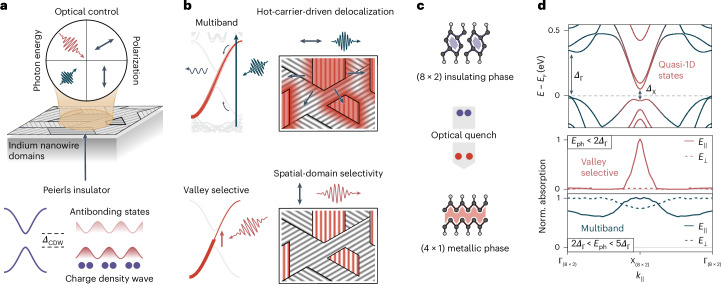
Optical surface electronic texture control via valley-selective photodoping. **a**, Top: schematic of the optical control parameters that drive selective surface domain switching. Bottom: the Peierls transition in a 1D atomic chain yields the formation of a periodic lattice distortion and CDW with a characteristic energy gap (*Δ*_CDW_). The electron density of valence (dark red) and conduction (light red) states exhibits a bonding and antibonding character with respect to the CDW along the chain direction. **b**, Top: light-induced electronic quench of the CDW phase. A population of states at the CDW gap, mediated by higher-lying surface bands (Multiband), is accompanied by electronic and lattice heating and yields a homogeneous phase change across the surface via delocalized energetic charge carriers. Bottom: direct bandgap excitation (Valley selective) minimizes dissipation, which manifests in domain-specific switching (red wires), with pronounced phase coexistence and local carrier confinement. **c**, Optical transition between the insulating (8 × 2) and metallic (4 × 1) phases of indium nanowires. **d**, Top: density-functional-theory-calculated electronic band structure of the Si(111) (8 × 2)–In phase (Methods). *Δ*_Γ_ and *Δ*_X_ denote the direct bandgaps, leading to the formation of energetic valleys in the band structure. Bottom: energy-integrated optical absorption. X-valley selectivity for low excitation energies and parallel polarization minimizes electronic heating and enables orientation-specific switching. Unspecific multiband absorption at higher excitation energies yields a delocalized hot-carrier distribution and homogeneous switching. The threshold energy is chosen to illustrate the transition between the regimes (see also Supplementary Fig. 1). *k*_∥_ is the momentum vector along the Brillouin-zone high-symmetry points with respect to the wire direction.

The interactions of electronic, orbital, spin and nuclear degrees of freedom govern the emergence of symmetry-broken functional states in solids. Light allows for tilting the balance between distinct states and phases^1,2^ and enables control over the final states by precisely tuning the optical interaction (Fig. 1a, top). A prototypical scenario is given by the optical quench of an electronic density modulation and lattice distortion in charge-density-wave (CDW) materials^3–6^. In the prevalent case of a Peierls insulator^7^, the interplay of quasi-one-dimensional (quasi-1D) electronic states at the Fermi energy and the lattice instability lead to the opening of a bandgap (*Δ*_CDW_) and the formation of electronic valleys in a metal–insulator transition (Fig. 1a, bottom)^8^. Photodoping of occupied bonding and unoccupied antibonding states at the band edges, in turn, collapses the bandgap and transiently reverses the phase change^9,10^. Although such optical switching frequently occurs on femtosecond timescales, it is largely indiscriminate with respect to the induced electronic transitions. In particular, electronic states near the gap are generally populated indirectly via rapid relaxation from optically accessible higher-lying bands (Fig. 1b, top)^3,11^. The deposited energy per lifted carrier, therefore, far exceeds that required for a minimally invasive phase transformation, which leaves the system with substantial lattice and electronic heat. A valley-polarized population from excitation at the bandgap^12^, on the other hand, may largely prevent dissipation to promote a minimum-energy transition pathway (Fig. 1b, bottom). Moreover, close to the photon-energy threshold, the phase change is expected to become particularly susceptible to local variations in the real-space microstructure and domain distribution^4,13^.

Realizing such conditions, in this work, we use the valley-selective photodoping of strongly coupled electronic states to demonstrate control over the domains and texture of a quasi-1D Peierls insulator. Specifically, we exploit the anisotropic absorption of nanowire domains by tuning both photon energy and polarization to the transition matrix elements most strongly coupled to the structural transformation. The reduction in electronic excess energy facilitates a minimum-energy pathway to domain-specific switching and decreases thermal relaxation. This selection of the carrier energy allows for spatial control over the phase transition, from delocalized carriers and homogeneous switching at high photon energy to locally confined carriers and domain-specific switching close to resonant gap excitation. The photoinduced formation of metallic nanodomains surrounded by insulating nanodomains demonstrated here represents the controlled preparation of nanoscale electronic textures, suggesting avenues for optically engineered electronic percolation and quantum confinement in ‘Peierls heterostructures’.

## Valley-specific excitation of Peierls-distorted atomic wires

Atomic wires formed by indium atoms on the (111) face of silicon^14^ are a prominent model system with near-ideal quasi-1D electronic properties. The corresponding surface reconstruction in a metallic (4 × 1) room-temperature phase is characterized by a parallel arrangement of atomic zigzag chains (Fig. 1c)^15^. Cooling below the critical temperature *T*_c_ = 125 K transforms the system into the insulating (8 × 2) hexagon phase in a triple-band Peierls transition^16^. A doubling of lattice periodicity is brought about by shear and rotary distortions, leading to the formation of interchain and intrachain covalent bonds within the coupled indium chains, respectively^17^. The phase change is equally reflected in the electronic band structure by the opening of bandgaps and concomitant formation of energetic valleys (Fig. 1d, top). Lateral shearing of zigzag chains causes a bandgap opening at the surface Brillouin-zone Γ point (*Δ*_Γ_)^18^, whereas the rotation-induced dimerization of outer indium atoms yields a bandgap at the X point (*Δ*_X_)^17^.

In particular, the formation of a supercooled metallic (4 × 1) phase can be triggered by light without reaching the phase transition temperature. In this process, optically generated electrons and holes rapidly scatter towards electronic states at the CDW gap, quenching the insulating bandgap and lifting the structural distortion on a 350-fs timescale^9^. Although the ultrafast transition involves a directed coherent nuclear motion driven by valley-specific couplings between carrier populations and well-defined unit-cell distortions^10,17,19^, the associated band structure dynamics were so far found to be independent of the incident photon energy^11,20^. However, the inherent anisotropy of bands at the insulating gap holds the potential for spatially distinct final-phase textures, which have not been explored^21^. We illustrate this anisotropy in the energy-integrated optical absorption of indium nanowires (Fig. 1d, bottom). At low photon energies, we find that absorption is largely limited to transitions in the X valley (at the insulating gap *Δ*_X_) and to a polarization parallel to the nanowire direction. By contrast, multiband absorption at higher excitation energies is unspecific towards strongly coupled states and exhibits a weaker perpendicular anisotropy.

## Creating coexistent electronic phases by polarization-selective switching

**Fig. 2:**
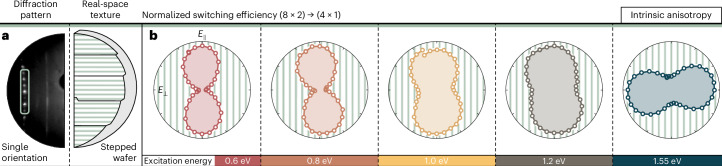
Polarization and photon energy dependencies of the (8 × 2) to (4 × 1) switching efficiency. **a**, LEED image and real-space sketch of parallel-oriented indium atomic wires on a stepped Si wafer surface with a 2° miscut relative to the (111) plane. **b**, Polarization-dependent switching efficiency for increasing photon energy, at the maximum diffraction spot suppression (1 – *I*(8 × 2)_Δ*t* = 40 ps_/*I*(8 × 2)_Δ*t* = −150 ps_). The in-plane electric-field component is depicted relative to the nanowire direction. Incident fluences (from left to right): 1.04 mJ cm^–2^, 0.59 mJ cm^–2^, 0.62 mJ cm^–2^, 1.09 mJ cm^–2^ and 1.40 mJ cm^–2^.

**Fig. 3:**
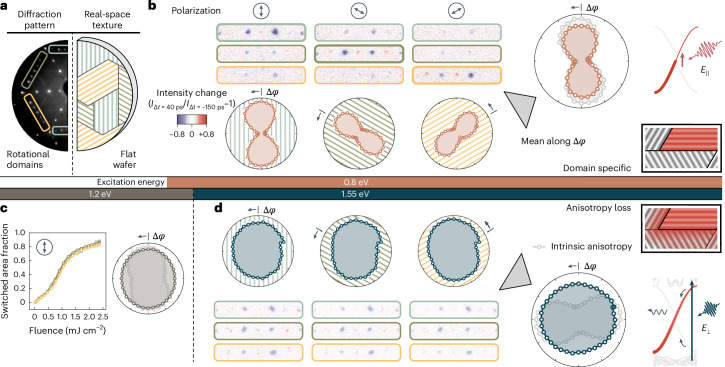
Polarization-sensitive and charge-transfer-induced switching in a domain texture. **a**, Three-fold symmetric rotational domains on a flat Si(111) wafer surface. The LEED image comprises a superposition of domain-specific reflexes. **b**, Polarization-dependent switching of rotational domains. Δ*φ* denotes the angle between the in-plane electric field and the nanowire orientation. **c**, Isotropic switching at near-IR wavelengths for all domain orientations in the entire fluence range. **d**, Transition between valley-selective to multiband excitation manifests in a loss of the intrinsic anisotropy (Fig. 2) due to the interdomain transfer of hot charge carriers. Incident fluences for 0.8 eV, 1.2 eV and 1.55 eV are 1.60 mJ cm^–2^, 1.40 mJ cm^–2^ and 2.03 mJ cm^−2^, respectively.

**Extended Data Fig. 1:**
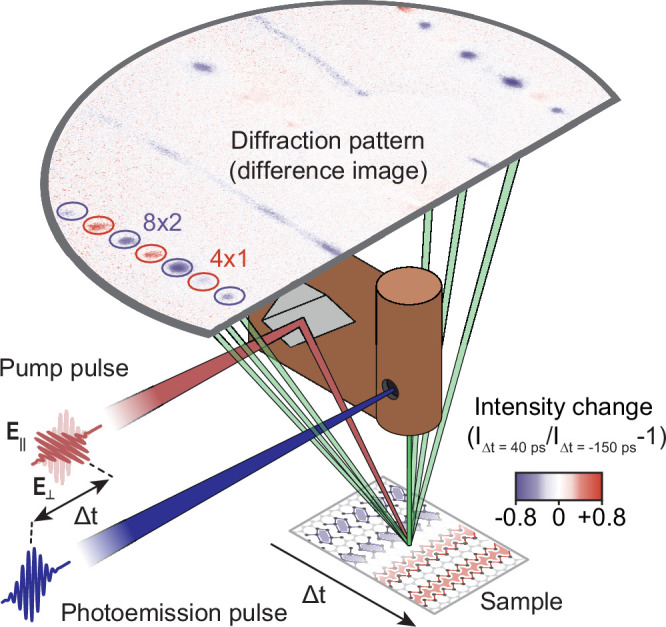
Experimental setup of ultrafast LEED. Ultrashort electron pulses, photoemitted from an electron gun, probe the microscopic surface structure after pump-probe delay *Δ*t. The optical quench of the structural distortion manifests in a loss/gain of (8 × 2)/(4 × 1) reflex intensity.

**Extended Data Fig. 2:**
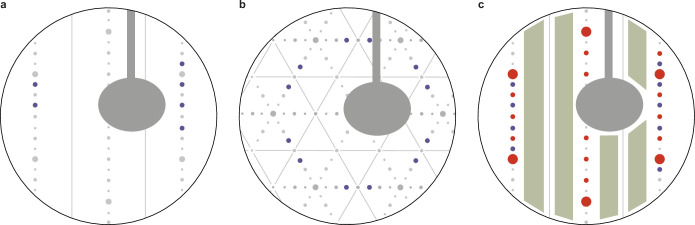
Analyzed diffraction features. Schematic diffraction image of the (8 × 2) indium phase on the (111) face of a Si wafer. (8 × 2) diffraction spots, analyzed in Fig. 2 on a stepped wafer (**a**) and in Fig. 3 on a flat wafer (**b**) are highlighted (blue). **c**, (8 × 2) (blue), (4 × 1) (red) and diffuse background (green) features, analyzed in Fig. 4.

**Extended Data Fig. 3:**
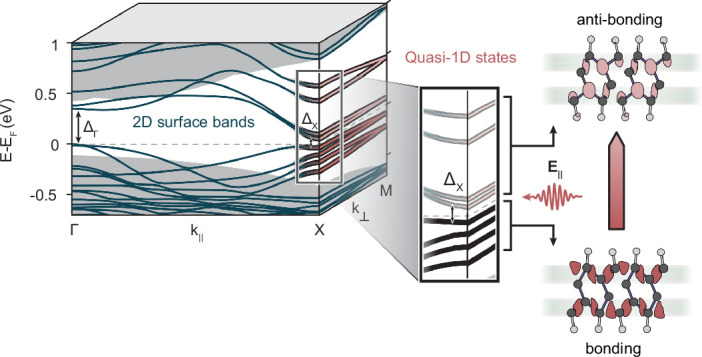
Band structure and valley-selective photoexcitation. Left, density-functional-theory (DFT) calculated electronic band structure of the Si(111)(8 × 2)-In phase (see Methods for details). Gray, projected Si bulk bands; dark green, surface localized bands; red, quasi-1D states with bonding and anti-bonding orbital character (right). *Δ*_*Γ*_ and *Δ*_X_ denote CDW gaps, leading to the formation of energetic valleys in the band structure. k_∣∣_ and k_⊥_ are the momentum vectors along Brillouin zone high-symmetry points with respect to the wire direction.

We explore this regime experimentally by using ultrafast low-energy electron diffraction (ULEED) combined with wavelength- and polarization-controlled excitation (Methods and Extended Data Fig. 1). In short, ULEED uses the backscattering diffraction of electron pulses from surfaces to probe optically induced changes in the atomic-scale structure^22–25^. In the present system, we monitor the phase transformation via changes in the intensity of characteristic diffraction peaks. Specifically, at a base temperature of *T* = 60 K, indium nanowires in the (8 × 2) CDW phase undergo a pump-induced ultrafast transition into the metastable (4 × 1) structure, which is subsequently probed by a photoemitted electron pulse. After the pump pulse, we observe an intensity decrease and increase in the (8 × 2) and (4 × 1) diffraction features, respectively. We systematically trace the photon-energy-resolved intensity suppression of the (8 × 2) diffraction features at a fixed time delay (Δ*t* = 40 ps) as a function of polarization, relative to the nanowire orientation (the analysed diffraction features are sketched in Extended Data Fig. 2). For this purpose, we differentiate two regimes of nanowire textures on the Si(111) surface: a single orientation, imposed by a linear step gradient on a 2°-miscut wafer (Fig. 2a) and rotational domains, formed naturally on the non-stepped surface (Fig. 3a) (domain-size range, 10^3^–10^4^ nm^2^ (ref. ^26^)). For a single orientation, we observe that the switching efficiency of the atomic wires is highly dependent on the incident electric-field polarization. At infrared (IR) driving wavelengths (Fig. 2b, left), the observed anisotropy aligns with the nanowire orientation, which specifically points towards direct absorption by quasi-1D electronic states close to the X point in the surface Brillouin zone (Fig. 1d, bottom). Here the coupling of the bonding and antibonding orbitals with respect to the CDW necessitates a polarization along the nanowire (Extended Data Fig. 3). This assignment is corroborated by the fingerprint of the involved electronic states, given by their preferential coupling to the vibrational rotation mode^10,17,19^. In consequence, optical excitation at the CDW gap yields a mode displacement that modulates the dimerization of indium atoms along the nanowire edges. Indeed, we observe an increased amplitude of the rotational mode in complementary coherent control experiments at IR wavelengths (Supplementary Note 1).

In the near-IR regime, the anisotropy gradually reverses towards perpendicular polarization (Fig. 2b, right), where the population of electronic states at the CDW gap is largely mediated by higher-energy surface bands, spread across the entire surface Brillouin zone. This reversal is intuitively explained by the energy-dependent alternation of Bloch function symmetries, which necessitates a rotation to perpendicular anisotropy at higher transition energies (Supplementary Note 2)^27^. Our findings are qualitatively consistent with linear reflectance anisotropy^21^ and IR spectroscopy^28^ studies, suggesting an intrinsic anisotropy due to polarization-sensitive absorption with respect to the nanowire orientation in conjunction with a nonlinear phase transition efficiency as a function of excitation density (Fig. 3c).

Harnessing the measured intrinsic anisotropy, we next demonstrate real-space texture control in the rotational domain structure (Fig. 3a). Here the nanowire orientation is directly linked to the diffraction reflex angle in the LEED image, providing orientation-resolved access to the phase change within the domain texture. To illustrate the dependence of the switching efficiency with the in-plane electric-field component, the domain-specific anisotropy is depicted with respect to the individual nanowire orientation (Fig. 3b). For IR excitation, the polarization dependence agrees with the intrinsic anisotropy, that is, with that measured on a stepped surface, which demonstrates domain-specific absorption and switching. At shorter near-IR wavelengths, in stark contrast to the intrinsic anisotropy, we find a surprisingly isotropic response (Fig. 3d), which is independent of the excitation density (Fig. 3c). From these data, we conclude that the homogeneous global response of all the domains stems from the delocalizing transfer of energetic photoexcited charge carriers across domain boundaries, effectively eliminating the intrinsic domain response. The strong dependence on photon energy is further attributed to the increasing amount of optical excess energy in the electron system after the insulator–metal transition, allowing for higher mobility across potential barriers between metallic and neighbouring insulating domains. A direct observation of this charge-transfer-induced phase transition in future studies could involve time-resolved scanning tunnelling microscopy^29,30^ or time- and angle-resolved photoemission spectroscopy.

## Suppressed ground-state relaxation from minimized thermal fluctuations

**Fig. 4:**
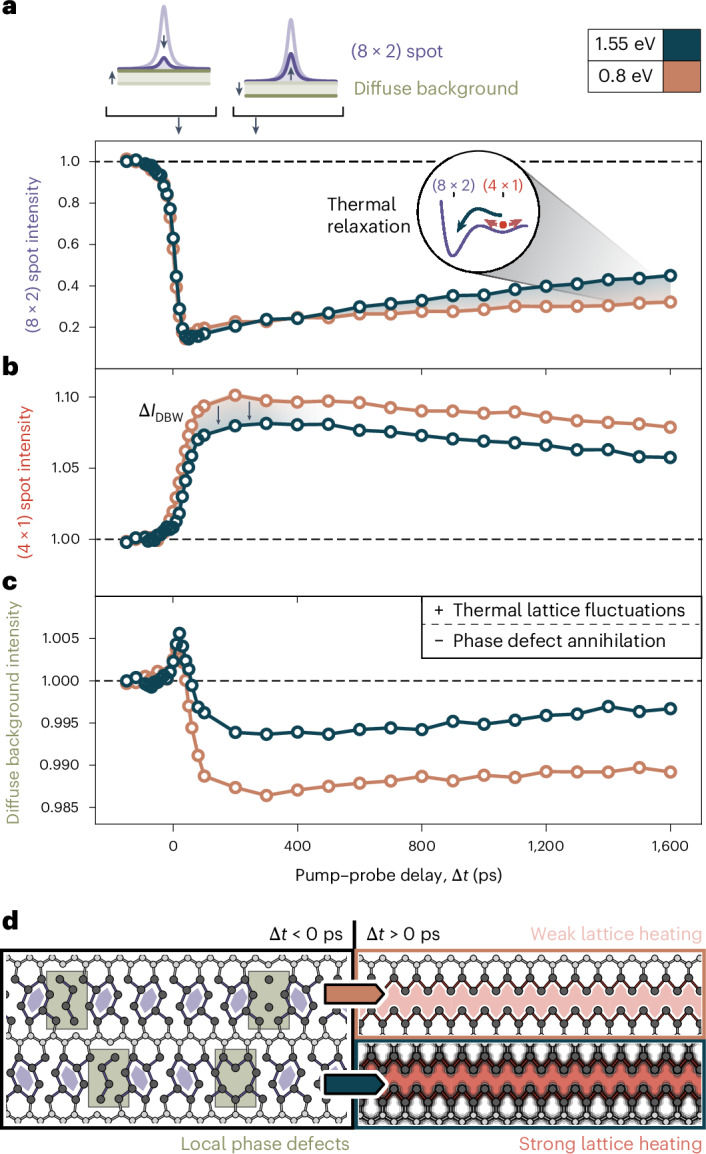
Lattice heating and thermal relaxation of the metastable (4 × 1) phase, driven by optical excess energy. **a**, Integrated (8 × 2) diffraction spot intensity as function of pump–probe delay Δ*t* at incident photon energies of 0.8 eV and 1.55 eV on a stepped wafer. The light polarization was chosen parallel and perpendicular to the nanowire orientation, respectively, corresponding to the maximum switching efficiency at fluences of *F*_0.8_ = 1.95 mJ cm^−2^ and *F*_1.55_ = 3.12 mJ cm^−2^. For identical intensity suppression, the relaxation into the (8 × 2) ground state accelerates with photon energy due to increasing thermal lattice fluctuations, following the phase transition (see the inset). **b**, Corresponding (4 × 1) diffraction spot intensity. Increased lattice heating at 1.55-eV excitation manifests in an intensity reduction due to the Debye–Waller effect (Δ*I*_DBW_). **c**, Pump-induced dynamic disorder increases the diffuse background intensity, counteracted by a reduction in the static disorder from enhanced phase homogeneity. **d**, Left: real-space sketch of indium atomic wires in the (8 × 2) phase, showing characteristic phase defects (alongside phase boundaries^46^), which cause diffuse background scattering. Right: the photoinduced (4 × 1) structure exhibits an increased phase homogeneity. Thermal lattice fluctuations cause an accelerated relaxation for increasing photon energy.

Beyond charge carrier confinement, we examine the implications of lattice heating from photon excess energy on the metastable state. Specifically, we track the temporal evolution of the (8 × 2) spot intensity at identical initial suppression on a stepped wafer and observe a prolonged recovery from near-IR to IR excitation (Fig. 4a). We ascribe this difference to photon-energy-dependent thermal fluctuations, driving the relaxation via over-the-barrier transitions (Fig. 4a, inset)^31^. This assignment is corroborated by a more pronounced suppression in (4 × 1) diffraction intensity at near-IR driving, due to a larger Debye–Waller effect at this excitation (Fig. 4b). The diffuse background intensity (Fig. 4c) provides further information on the degree of dynamic and structural disorders of the surface^32^. The photon-energy-dependent thermal fluctuations are reflected in different levels of the diffuse background throughout the delay range. In addition, however, both curves display a global reduction in the diffuse background below the value before excitation. We interpret this finding as direct evidence for a laser-induced ordering of the surface on the transition to the metastable (4 × 1) phase. Specifically, the annihilation of localized defects in the CDW lattice^33,34^ render this surface more long-range ordered. Besides a potential narrowing of the spot widths, which could not be resolved experimentally, the absence of such point-like defects in the (4 × 1) phase will also decrease diffuse scattering throughout the Brillouin zone. Our findings, thus, imply that valley-selective excitation approaches a minimum-energy pathway for the phase transition and leaves the system in a highly ordered state from which thermal relaxation is suppressed (Fig. 4d).

## Discussion and outlook

From these observations, an intuitive picture of the spatiotemporal phase-change dynamics in the regime of valley-selective versus multiband excitation emerges. IR photon absorption effectively confines charge carriers to rotational nanodomains, selected by the incident polarization. The population and depopulation of strongly coupled electronic states at the X valley collapses the bandgap (Fig. 1d, *Δ*_X_) in an insulator–metal transition within about 200 fs (ref. ^11^), localizing the low-energy photoexcited charges to the absorbing domain. As lattice fluctuations are suppressed, thermal ground-state recovery slows down on the nanosecond timescale^25,35^. On the other hand, near-IR absorption across the band structure yields a delocalized hot charge carrier distribution, homogeneous switching and pronounced lattice heating.

**Extended Data Fig. 4:**
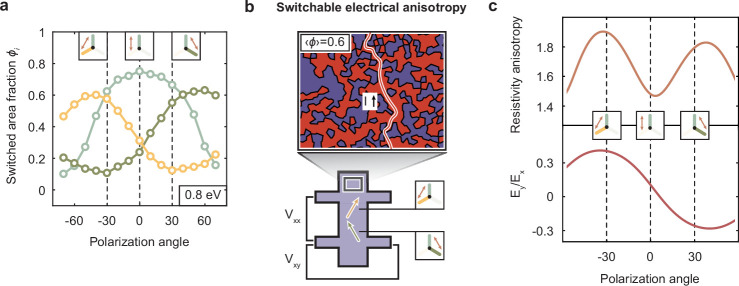
Predicted functionality of an optically switchable percolation network. **a**, Measured rotational domain-resolved surface area fraction, switched into the (4 × 1) phase at 0.8 eV photon energy (see also Fig. 3b). The insets indicate particularly domain-selective optical polarization states. **b**, Proposed optical steering of electronic transport in a percolated domain texture within a Hall bar geometry. Red, metallic (4 × 1); blue, insulating (8 × 2) domains. Based on the anisotropic conductivity of indium atomic chains, selective domain switching could be used to control the transversal charge flow within the metallic percolation network. **c**, Top, calculated anisotropy along the conducting network principal axes with incident polarization, using experimental data. The anisotropy in resistance along the principal axes is peaked when one orientation is deselected by choosing a polarization perpendicular to the wire direction. In this scenario, the preferential transport along the wire direction yields a lateral deflection of charge carriers, resulting in a transversal voltage with tunable polarity. Bottom, transversal to longitudinal voltage ratio across the Hall bar. The voltage polarity can be optically switched by deselecting individual wire orientations.

**Extended Data Fig. 5:**
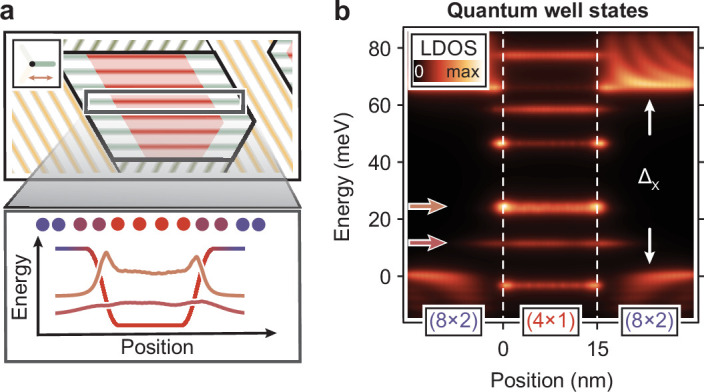
Tight-binding calculation of Peierls heterostructure local density of states. **a**, Sketch of transient quantum well formation due to suppressed relaxation dynamics and domain-specific switching. Long-lived metallic segments are embedded in relaxed insulating nanowires. **b**, Tight-binding calculations of the local density of states along the atomic chain show the formation of quantized metallic states within the insulating band gap (*Δ*_X_). In the transition region one expects strong band bending and therefore high curvature, which increases the effective mass of the electron and causes localization. Mismatch in band alignment between the metallic and insulating phase further leads to confinement even beyond the insulating gap in metallic and insulating segments.

We believe that optically engineered phase textures expand the paradigm of light-induced phenomena and enable a polarization-sensitive control of functional states in real space. Therefore, motivated by our experimental findings, we theoretically explore a tangible implementation of ‘Peierls heterostructures’. Polarization-specific switching can yield optically controlled electronic properties in percolation networks (Extended Data Fig. 4 and Supplementary Note 3 provide the proposed device design). Although homogeneous switching results in a surface texture with isotropic conductivity, that is, an equal fraction of metallic nanowire orientations, domain-specific switching promises tunable anisotropy when the incident polarization and fluence are adjusted accordingly. Furthermore, a possible implementation of optically tailored electronic properties is given by the transient formation of isolated metallic grains at parallel polarization, resulting in the quantization of electronic states within the insulating bandgap (Extended Data Fig. 5 and Supplementary Note 2 show the tight-binding simulations of the the resulting local density of states).

In conclusion, our results demonstrate the quench of a Peierls insulator by means of valley-selective optical transitions that specifically address bonding and antibonding orbitals. In this way, we achieve deep subwavelength precision in an optically driven phase change and gain control over the real-space texture. Our results suggest an important role of optical excess energy in photoinduced phenomena with immediate ramifications for the ability to prepare and manipulate coexisting electronic phases. The inherent nature of Peierls physics at the core of this quasi-1D system implies a broader relevance to materials, where changes between electronic phases are instigated by distinct low-symmetry electronic states. As such, we believe that polarization-sensitive switching offers a versatile framework for patterning domain structures and controlling nanoengineered functionalities across various exotic electronic systems, such as correlated oxides^4,13,31,36–39^, Weyl semimetals^40,41^ and materials exhibiting electronic nematicity^42–45^. Hence, the optical preparation of electronic phase separation introduces an additional degree of freedom in light-induced switching.

## Methods

### Density functional theory calculations

We performed density functional theory simulations within the local density approximation^47^ as implemented in the Vienna ab initio simulation package^48^. The electronic structure is described by projector-augmented wave potentials^49^ with a plane-wave basis set limited to a cut-off energy of 250 eV. The surface was modelled using periodic boundary conditions and a slab with three bilayers of silicon. Si dangling bonds at the bottom layer were saturated with hydrogen. To determine the ground-state electronic structure, a 2 × 8 × 1 Monkhorst–Pack mesh was used to sample the Brillouin zone of the Si(111)–(8 × 2)In structure, corresponding to 256 *k* points in the Si(111)–(1 × 1) surface unit cell. The band structure calculations were performed using 40 (10) *k* points along the Γ–X (X–M) direction.

The orbital character of the bonding and antibonding states (Extended Data Fig. 3) was determined by integrating the electronic density of states along the X–M direction and across the topmost four valence bands and lowest four conduction bands, respectively. The resulting densities are plotted in Extended Data Fig. 3 at an isovalue of 0.033 e^−^ Å^−3^.

Consistent with the numerical approach outlined elsewhere^21^, the oscillator strength was calculated in independent particle approximation from the squared transition matrix elements (∥*M*_*if*_∥^2^) between all the occupied (*i*) and unoccupied (*f*) surface bands within the Brillouin zone along the depicted high-symmetry points at parallel and perpendicular polarizations with respect to the wire direction. To this end, we considered 240 occupied and 120 empty bands. The calculated values at all the momenta are energy integrated and normalized to the overall maximum.

### Ultrafast LEED and optical setup

Ultrafast LEED is a technique for the investigation of structural dynamics at solid-state surfaces by means of an optical pump and electron probe scheme (Extended Data Fig. 1)^22–25^. It combines the high surface sensitivity of photoemitted low-energy electrons in backscattering geometry with ultrafast optical excitation to follow the evolution of non-equilibrium surface structures .

The demonstrated high temporal and momentum resolutions are achieved using a custom-built laser-driven electron gun consisting of a nanometric tungsten tip as well as four metal electrodes (outer diameter, 2 mm). Ultrashort electron pulses are generated from localized two-photon photoemission by illuminating the tip apex with femtosecond laser pulses (central wavelength, *λ*_c_ = 400 nm; pulse duration, *τ*_p_ = 45 fs; pulse energy, *E*_p_ = 30 nJ) at a repetition rate of 100 kHz. The small diameter of the electron gun allows for a small sample distance without blocking the backscattered electrons, resulting in pulse durations down to 16 ps. Detected electrons are amplified and recorded by a combination of a chevron microchannel plate, a phosphor screen and a scientific complementary metal–oxide–semiconductor camera.

In the described experiments, the surface is excited using a wavelength-tunable optical pump pulse from an optical parametric amplifier (*λ*_c_ = 800 nm, 1,240 nm, 1,550 nm and 2,066 nm; *ℏω* = 1.55 eV, 1.0 eV, 0.8 eV and 0.6 eV; Δ*τ* = 232 fs) or with a fixed pulse wavelength from a Yb:YAG amplifier system (*λ*_c_ = 1,030 nm, *ℏω* = 1.2 eV and Δ*τ* = 212 fs). The surface is probed by a photoemitted 80-eV electron pulse with a beam diameter of ~80 × 80 μm^2^ (full-width at half-maximum).

### Sample preparation

All the experiments in this work have been conducted under ultrahigh-vacuum conditions (base pressure, *p* < 2 × 10^−10^ mbar), thereby minimizing the effect of adsorbate-related (8 × 2) ground-state recrystallization from the supercooled (4 × 1) phase^50^. The silicon substrate (phosphorus-doped wafers with resistivity *R* = 0.6–2 Ω cm), showing only a single-wire orientation, was miscut by 2° towards the [–1 –1 2] direction to create a high surface step density. The oriented steps effectively confine the atomic indium wires along one crystallographic direction. On the other hand, wafers cut along the (111) face of silicon exhibit much larger terraces, where all the three crystallographically equivalent orientations are equally found. All the samples were cleaned by flash annealing at 1,350 °C for 5 s via direct-current heating. Subsequently, 1.2 monolayers of indium were deposited onto the resulting Si(111) (7 × 7) surface reconstruction at room temperature and annealed at *T* = 400 °C for 300 s. The resulting Si(111) (4 × 1)–In phase was inspected in our ultrafast LEED setup and subsequently cooled to a base temperature of *T* = 60 K using an integrated continuous-flow helium cryostat. The phase transition between the high-temperature (4 × 1) and low-temperature (8 × 2) phases was observed at a temperature of 125 K.

## Online content

Any methods, additional references, Nature Portfolio reporting summaries, source data, extended data, supplementary information, acknowledgements, peer review information; details of author contributions and competing interests; and statements of data and code availability are available at 10.1038/s41567-025-02899-5.

## Supplementary information

Supplementary Information Supplementary Figs. 1–8 and Notes 1–3.

## Data Availability

The data shown in the Article are available via Edmond (the Open Research Data Repository of the Max Planck Society) at 10.17617/3.AIYWA4 (ref. ^51^).
