# Supplementary material for: Valley-controlled photoswitching of metal–insulator nanotextures
Source: Nat Phys. 2025 May 21;21(7):1106–11. doi: 10.1038/s41567-025-02899-5 (PMC12263438; doi:10.1038/s41567-025-02899-5)
Supplement: Supplementary file 1 — Supplementary Figs. 1–8 and Notes 1–3. [file 41567_2025_2899_MOESM1_ESM.pdf]

nature physics

Supplementary information

https://doi.org/10.1038/s41567-025-02899-5

Valley-controlled photoswitching of metal–insulator nanotextures

In the format provided by the authors and unedited

Supplementary information

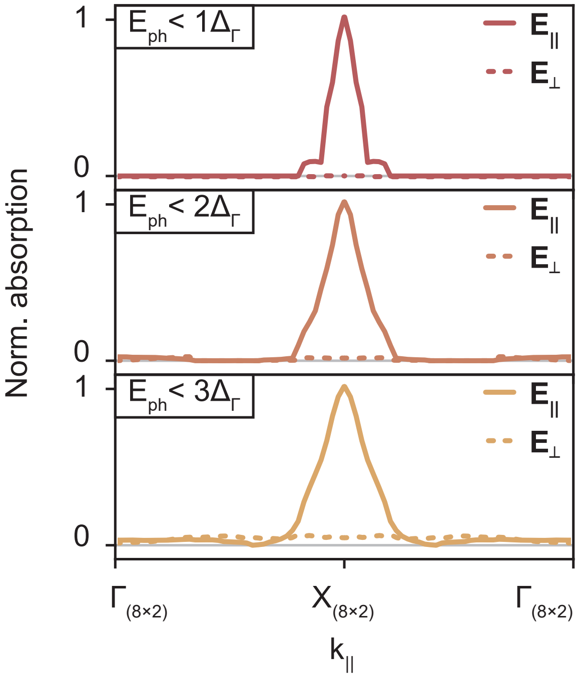

Supplementary Fig. S1: DFT-calculated normalized optical absorption. In analogy to Fig. 1 of the main text, we show the energy-integrated and normalized oscillator strength in different photon energy regimes. A pronounced parallel anisotropy and localization of the absorption at the X-valley persists for energies, far exceeding the band gap at the Γ-point (Δ_Γ).

Supplementary Note 1: Coherent control at IR- and near-IR excitation

A direct fingerprint of the involved electronic states is given by their preferential coupling to the individual amplitude phonon modes that modulate the structural distortion upon excitation [1, 2, 3]. Specifically, DFT calculations show that the electronic rearrangement due to the population of states at the Γ-point initiates a shearing between coupled indium chains while the depopulation of states around the X-point drives a hexagon rotary motion (Fig. S2a). We detect the resulting structural dynamics from an optical excitation in a coherent control scheme [4], where a weak first pulse of variable wavelength creates vibrational coherence within the (8×2) minimum of the potential energy landscape along the structural distortion (Fig. S2b). Subsequently, a stronger near-infrared pulse transforms a fraction of indium atomic wires into the (4×1) phase, as probed by an electron pulse at Δt=40 ps. The switched surface fraction thus becomes dependent on the momentary vibrational state and its impact on overcoming the potential energy barrier [3]. Oscillations in the relative switching yield with delay time between optical pulses reveal the vibrational modes, excited by 1.0 eV photons (Fig. S2c). A Fourier transform shows contributions from both shear and rotary modes, as previously observed for higher photon energies [4], suggesting relevant transitions within the entire surface Brillouin zone (Fig. S2d) [5]. The relative strength of the shear mode is likely attributed to the stronger electron-phonon coupling of zone-center electronic states [2]. On the other hand, spectral transitions from 0.6 eV photons are highly localized at the X-point, thus coupling primarily to the rotation mode, as manifested in the oscillatory component of the relative switching yield (Fig. S2e) and the corresponding Fourier transform (Fig. S2f). The inferred decisive role of charge carriers around the X-point for the phase transition is in perfect agreement with photoemission spectroscopy and molecular dynamics simulations [5].

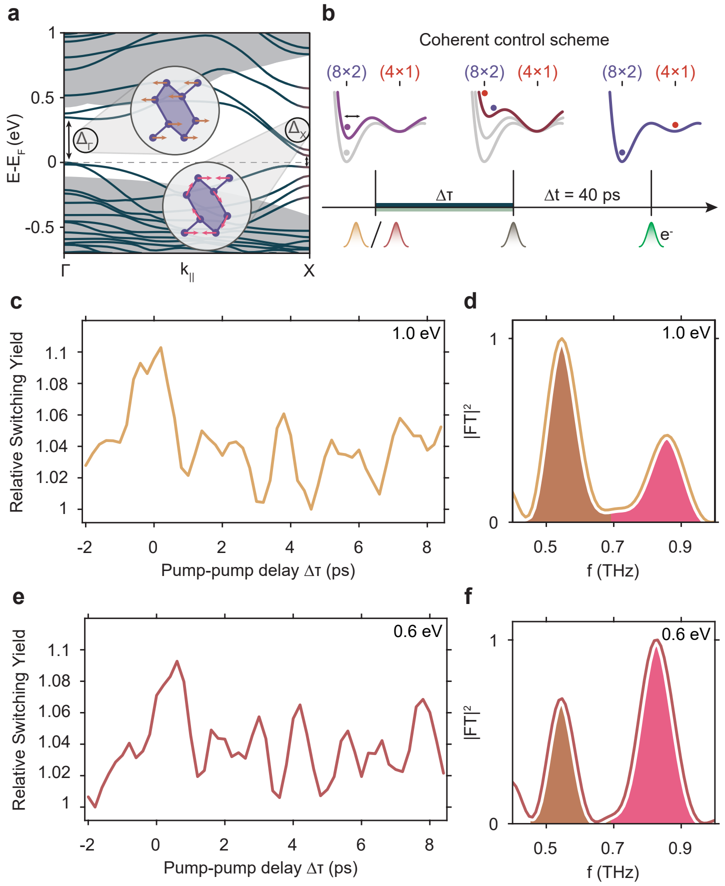

Supplementary Fig. S2: Photon-energy-resolved vibrational coherences in coherent control measurements. (**a**), Calculated band structure along the momentum direction, parallel to the atomic wire. Band gaps at the Γ- and X-points preferentially couple the shear and rotation vibrational amplitude modes, respectively. (**b**), Scheme for coherent control measurements. A first weaker preparation pulse creates a coherent vibrational oscillation in the ground state (left), which is translated into a measurable difference in ensemble averaged switching efficiency, depending on the momentary vibrational state upon the arrival of a stronger switch pulse (E_{ph}=1.2 eV) after time $\Delta\tau$ (center). The resulting switched surface fraction is probed by a later electron pulse at Δt=40 ps (right). (**c**), Relative switching yield as function of $\Delta\tau$ for a preparation pulse photon energy of 1.0 eV, showing characteristic vibrational oscillations. (**d**), The corresponding Fourier transform reveals the relative amplitudes at the shear mode (0.55 THz) and rotation mode frequencies (0.82 THz) [1, 6]. (**e**), Analogous measurement for a preparation pulse photon energy of 0.6 eV (**f**), The Fourier transform of the time trace shown in (**e**).

Supplementary Note 2: Tight-binding simulations

Model for homogeneous systems

Following Ref. [2] we choose a quasi-1D tight-binding Hamiltonian with four coupled parallel chains. Indexing of the lattice sites for two (4×1) unit cells is shown in Fig. S3. Due to the symmetry in this setup we denote this as the symmetric configuration or metallic phase (see the discussion further below on the band structure of the system). Note that this configuration corresponds to the high temperature phase. We will add a distortion to the lattice, which leads to a metal-insulator transition and doubled lattice periodicity, similar to a Peierls transition [7]. This corresponds to the (8×2) low-temperature configuration.

We discuss our approach on the simpler symmetric configuration, which consists of 4 sites per unit cell. Let $m \in \{1, ..., N\}$ be the index for the unit cell and $\alpha \in \{A, B, C, D\}$ the intracell index. A general tight-binding Hamiltonian in real space is denoted as:

$$H = \sum_{(m,\alpha),\sigma} \epsilon_{(m,\alpha)} c^{\dagger}_{(m,\alpha),\sigma} c_{(m,\alpha),\sigma} - \sum_{<i,j>,\sigma} t_{ij} \left(c^{\dagger}_{i,\sigma} c_{j,\sigma} + c^{\dagger}_{j,\sigma} c_{i,\sigma} \right) , \quad (1)$$

where σ is the spin index, $\epsilon_{(m,\alpha)}$ is the on-site energy on lattice site (m, α), and t_{ij} is the hopping strength between lattice sites i and j, where we simplify the notation by setting $(m, \alpha) =: i$ and $(m', \alpha') =: j$ in the second sum. $c^{(\dagger)}_{i,\sigma}$ is the usual fermionic annihilation (creation) operator for an electron with spin σ on lattice site i. We omit the spin index of the two spin directions in the following, as they are treated independently. In position space, this leads to the following matrix representation of the Hamiltonian:

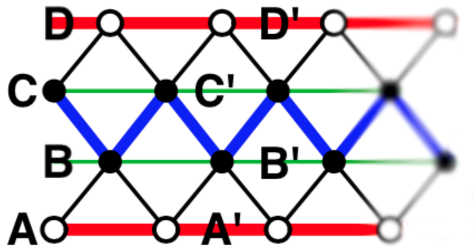

Supplementary Fig. S3: Lattice site indexing for two unit cells in the symmetric (high-temperature) configuration. [modified from Ref [2]].

$$H_{\text{reduced}} = \left(\begin{array}{cccc|cccc} \epsilon_{AA} & t_{AB} & t_{AC} & t_{AD} & t_{AA'} & t_{AB'} & t_{AC'} & t_{AD'} \\ t_{BA} & \epsilon_{BB} & t_{BC} & t_{BD} & t_{BA'} & t_{BB'} & t_{BC'} & t_{BD'} \\ t_{CA} & t_{CB} & \epsilon_{CC} & t_{CD} & t_{CA'} & t_{CB'} & t_{CC'} & t_{CD'} \\ t_{DA} & t_{DB} & t_{DC} & \epsilon_{DD} & t_{DA'} & t_{DB'} & t_{DC'} & t_{DD'} \\ \hline t_{A'A} & t_{A'B} & t_{A'C} & t_{A'D} & \epsilon_{A'A'} & t_{A'B'} & t_{A'C'} & t_{A'D'} \\ t_{B'A} & t_{B'B} & t_{B'C} & t_{B'D} & t_{B'A'} & \epsilon_{B'B'} & t_{B'C'} & t_{B'D'} \\ t_{C'A} & t_{C'B} & t_{C'C} & t_{C'D} & t_{C'A'} & t_{C'B'} & \epsilon_{C'C'} & t_{C'D'} \\ t_{D'A} & t_{D'B} & t_{D'C} & t_{D'D} & t_{D'A'} & t_{D'B'} & t_{D'C'} & \epsilon_{D'D'} \end{array}\right) =: \left(\begin{array}{c|c} H_{\text{cell}}^{\text{intra}} & T_{\text{cell}}^{\text{inter}} \\ \hline (T_{\text{cell}}^{\text{inter}})^\dagger & H_{\text{cell}}^{\text{intra}} \end{array}\right) \tag{2}$$

Note that this matrix considers only intercell hopping between two adjacent unit cells as well as intracell hopping inside the two unit cells. The hopping rates t_{ij}, which are finite for our system, are colour coded (equal colors correspond to equal transition amplitudes in the symmetric case). In the second equality we have split up the Hamiltonian into intercell and intracell operators. Given the shape of the unit cell, we can write the Hamiltonian as a tensor product:

$$H = \sum_n |n\rangle\langle n| \otimes H_{\text{cell}}^{\text{intra}} + \left(\sum_n |n\rangle\langle (n \bmod N) + 1| \otimes T_{\text{cell}}^{\text{inter}} + h.c.\right) \tag{3}$$

The n mod N term implements the periodic boundary conditions in the direction parallel to the wires (in perpendicular direction, we apply open boundary conditions). We use the discrete translational symmetry of the system to calculate the momentum eigenstates using $|n\rangle = \frac{1}{\sqrt{N}} \sum_k e^{-ikn} |k\rangle$ with $k = \frac{2\pi m}{aN}$ and $m = 1, ..., N$ and set the distance between two unit cells $a = 1$. Note that k here corresponds to $k_\parallel$ in Fig. 1d of the main text. Following the naming of transition variables from Ref. [2], we obtain the Bloch Hamiltonian $H(k)$ for the (4×1) unit cell:

$$H(k) = \begin{pmatrix} \epsilon_O - 2t_O \cos k & -2t_{IO} e^{\frac{-ik}{2}} \cos \frac{k}{2} & 0 & 0 \\ -2t_{IO} e^{\frac{ik}{2}} \cos \frac{k}{2} & \epsilon_I - 2t_{I2} \cos k & -2t_{I1} e^{\frac{ik}{2}} \cos \frac{k}{2} & 0 \\ 0 & -2t_{I1} e^{\frac{-ik}{2}} \cos \frac{k}{2} & \epsilon_I - 2t_{I2} \cos k & -2t_{IO} e^{\frac{-ik}{2}} \cos \frac{k}{2} \\ 0 & 0 & -2t_{IO} e^{\frac{-ik}{2}} \cos \frac{k}{2} & \epsilon_O - 2t_O \cos k \end{pmatrix} \tag{4}$$

The eigenstates of the entire system are then found via numerical diagonalization of $H(k)$ and calculation of the tensor product with $|k\rangle$ for each allowed value of k. For each energy eigenvector with $H|\Psi_n\rangle = E_n|\Psi_n\rangle$ we find a corresponding state, constructed from $|k\rangle$, and the eigenvectors of $H(k)$, i.e. $|\Psi_n\rangle \leftrightarrow |k\rangle \otimes |i\rangle$ (up to an arbitrary complex phase), where $|k\rangle = \frac{1}{\sqrt{N}} \sum_n e^{-ikn} |n\rangle$ and $|i\rangle$ are the eigenvectors of $H(k)$. Due to the dimension of $H(k)$ we have four energies for each possible value of k, corresponding to the band structure for the symmetric configuration (Fig. S4a). We adapt the parametrization from Ref. [2] to model

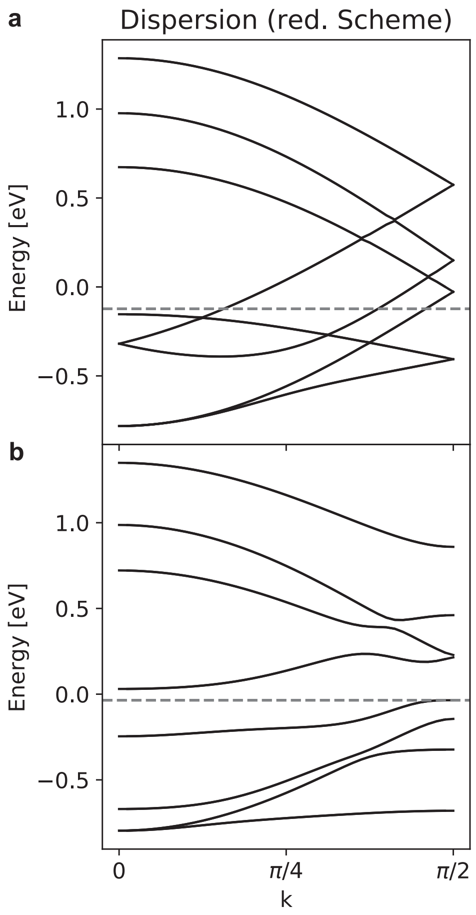

Supplementary Fig. S4: Electronic band structure for (**a**) the metallic symmetric phase and (**b**) the distorted insulating phase. Bands are folded back around the X-point of the distorted configuration. The respective Fermi energies are shown as a dashed line in both plots.

the metal to insulator phase transition of the indium nanowires. To this end, two parameters are introduced, corresponding to shear- and rotational structural distortions. The effect on the transition rates is then modeled by:

$$t_{ij}(d_{ij}) = t_{ij} \exp\left[-\alpha_{ij}\left(d_{ij} - d_{ij}^0\right)\right], \tag{5}$$

where d_{ij}^0 and d_{ij} are the distances between lattice sites i and j before and after distortion, respectively. The distortion leads to a unit-cell doubling due to discrete translational symmetry breaking. Fig. S4b shows the band structure of the symmetry-broken configuration in a reduced scheme, i.e. the band structure is folded back around the X-point.

Current operator

In order to discuss the currents and the associated absorption anisotropies, we treat the system in the two-dimensional plane with coordinates (x, y) and unit vectors $\hat{x}$ and $\hat{y}$ for both directions, respectively. We can then define currents in the system, which are parallel to the chain directions (longitudinal direction, denoted by $\hat{x}$) and orthogonal to it (transverse direction, denoted by $\hat{y}$). Following Ref. [8], we can formulate a current operator for each direction:

$$J_x = \frac{N_\perp}{Na} \sum_{ij} t_{ij}(d_{ij}) \left(\hat{t}_{ij} \cdot \hat{x}\right) \left(c_i^\dagger c_j - c_j^\dagger c_i\right) \,, \tag{6}$$

where $\hat{t}_{ij}$ is the unit vector pointing in the direction of the bond connecting lattice sites i and j and $N_\perp$ is the number of chains perpendicular to the chain direction, i.e. four in this model. Analogously, for the orthogonal direction we replace $\hat{x}$ by $\hat{y}$. We use the expression for the optical conductivity derived in Ref. [9] and adapt it to account for half-filling. In this way, we calculate the absorption spectrum as the real part of the optical conductivity $\sigma(\omega)$:

$$\sigma_{x/y}(\omega) = \frac{\pi}{\omega} \operatorname{Im} \left[\sum_{\substack{n_0 \leq \frac{N}{2} \\ n_{\mathrm{ex}} > \frac{N}{2}}} \left| \langle \Psi_{n_0} | \hat{J}_{x/y} | \Psi_{n_{\mathrm{ex}}} \rangle \right|^2 \left(\frac{1}{\omega + (E_{n_{\mathrm{ex}}} - E_0) + i\gamma} - \frac{1}{\omega - (E_{n_{\mathrm{ex}}} - E_0) + i\gamma} \right) \right] \tag{7}$$

Optical conductivities

In the presented tight-binding calculations, the complexity arising from time-dependent optical intensity or the manifold of structural degrees of freedom acting in concert, is omitted. However, a qualitative understanding of valley-selective optical absorption, arising from the formation of a CDW gap is captured by Eq. 2. We calculate the optical conductivities from optical transitions in the band structure for both the symmetric and distorted nanowire structures (Fig. S5). The current operator matrix elements $|\langle \Psi_{n_0} | \hat{J}_{x/y} | \Psi_{n_{\mathrm{ex}}} \rangle|^2$ of individual transitions are depicted in Fig. S5b and e, while optical conductivities for both phases are shown in Fig. S5c and f. In qualitative agreement with DFT calculations (see Fig. 1d of the main text), we find a strongly anisotropic absorption for the insulating phase at the zone-boundary CDW gap along the nanowire direction.

Model for a Peierls heterostructure

The Hamiltonian for the Peierls heterostructure combines the insulating Peierls-distorted phase and the metallic symmetric phase. We model the Hamiltonian as being made up of (8×2) unit cells where the distortion parameters for each cell can be individually altered and the transition rates change according to Eq. 5. A metallic domain of 20 cells width $\approx 15.4\,\mathrm{nm}$ [2] is

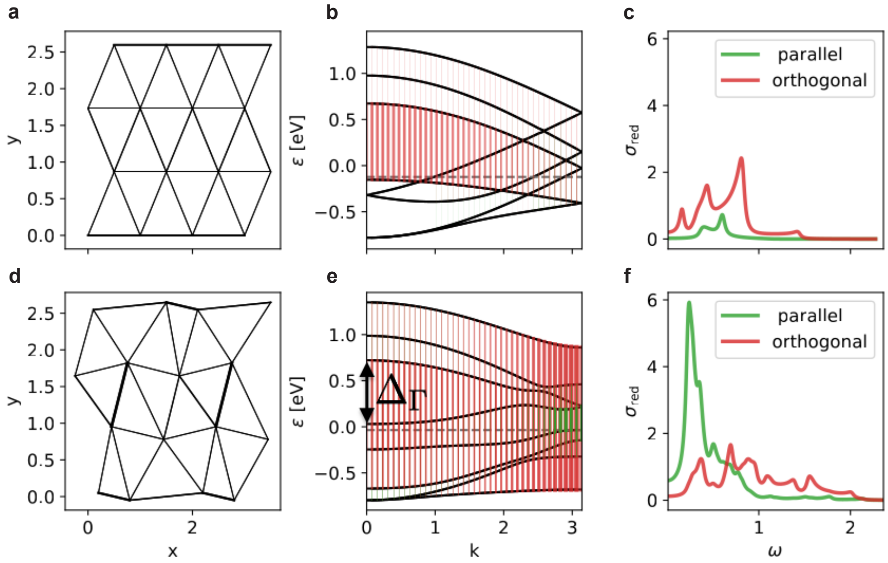

Supplementary Fig. S5: Tight-binding simulation results for homogeneous systems. (**a, d**) Lattice configuration in real space, (**b, e**) band structure with weighted transitions and (**c, f**) optical conductivities for the metallic phase and insulating phase, respectively. Δ_Γ corresponds to the energy gap in Fig. 1d of the main text.

sandwiched between two equally sized homogeneously distorted insulating regions (each of 96 cell width $\approx 73.7\,\mathrm{nm}$). Given the periodic boundary conditions in our model, the two insulating regions should be considered as one region connecting to the metallic domain on both sides. As a side note, the cells in the metallic region contain two symmetric unit cells, i.e. 40 (4×1) unit cells. Large jumps in the distortion parameters on the phase junction/boundary are avoided by designating a transition region of 4 cells ($\approx 3.1\,\mathrm{nm}$) where the parameters are linearly interpolated (Fig. S6). This junction size is derived from experimentally determined values of 0.4-7 nm [10].

The corresponding Hamiltonian is not symmetric under arbitrary lattice translations, hence defining crystal momentum in this system is non-trivial. Instead of investigating the band structure, we calculate the local density of states (LDOS) which we define at cell index i and energy E as $\mathrm{LDOS}(i, E) := \sum_{n,x\,\in\,\mathrm{unitcell}(i)} |\Psi_n(x)|^2\,\delta(E - E_n)$. Since we are grouping 8 sites of a unit cell into one cell index, using the cell index to be the position is only valid as long as the distortion of the unit cells is small. This is the case for our choice of parameters. We broaden the resulting LDOS values with a bivariate Lorentzian with a mean absolute deviation of $\gamma = 0.015$, corresponding to a spatial resolution of approximately 1.5 nm and $3 \cdot 10^{-3}$ eV,

$$\mathrm{LDOS}_\gamma(i, E) = \sum_{l,n} \frac{\gamma \cdot \mathrm{LDOS}(l, E_n)}{2\pi \left(\left(\frac{i-l}{N}\right)^2 + \left(\frac{E-E_n}{E_{\max}-E_{\min}}\right)^2 + \gamma^2 \right)^{3/2}} \,. \tag{8}$$

The calculated LDOS for the Peierls heterostructure is depicted in Extended data Fig. 5.

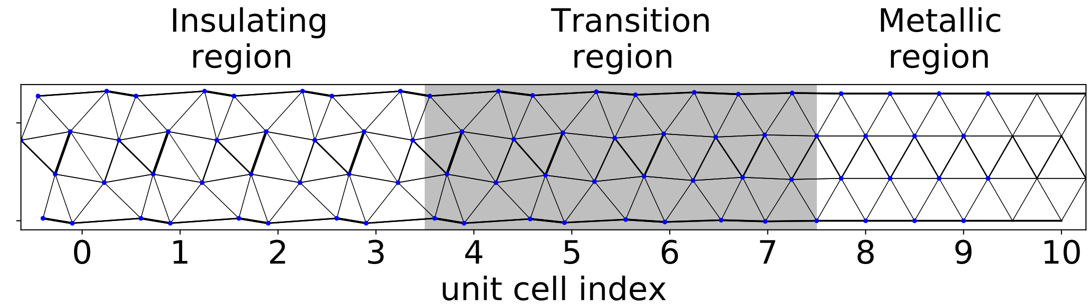

Supplementary Fig. S6: Lattice for a heterostructure with a transition region extending over 4 unit cells.

Supplementary Note 3: Optical switching of electrical anisotropy in a percolation network

Conductivity in a percolation network

The rotational domain texture of indium nanowires [11] is approximated by a randomized Voronoi tiling. To this end, a Voronoi network and corresponding Delauney triangulation is constructed from a Poisson-distributed point grid of 600×600 sites. For finding the percolation threshold, we follow established procedures [12, 13].

An active seed site is placed on the network center and its neighbors are turned on with a probability p or turned off with probability $1 - p$ [15]. Depending on p, the seed will either die off or grow indefinitely, thus marking the transition towards a fully percolated network. Statistical averaging was performed across 500 runs for each p to determine the critical threshold p_c for growth beyond the grid bounds (Fig. S7a). We find $p_c \approx 0.54$, in good agreement with the literature value for Voronoi tiling percolation ($p_c = 0.5$) [12, 13]. Above the percolation threshold, p approaches the network filling factor ϕ, i.e. the metallic/insulating surface fraction. The effective conductivity of the network, in this regime, follows a universal scaling law [14]:

$$\sigma_{eff} \propto (\langle \phi \rangle - \phi_c)^t \tag{9}$$

where t is a critical exponent with universal value of 1.3 for 2D systems (Fig. S7b) [16]. For the presented simulations, we chose $\langle \phi \rangle = 0.6$, above the percolation threshold.

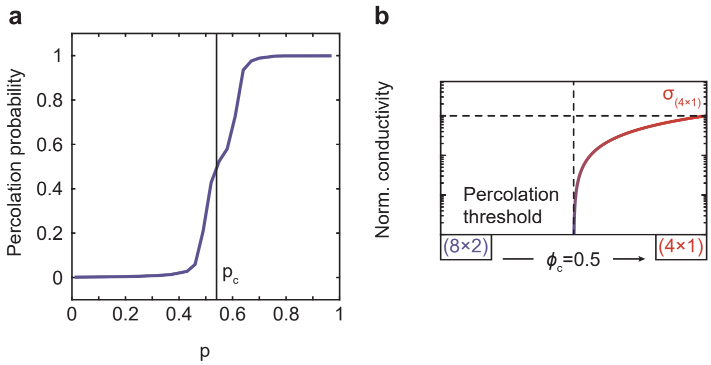

Supplementary Fig. S7: Voronoi tiling percolation and conductivity in percolation network. (**a**), The size of the largest connected component in a randomized Voronoi network is calculated as function of switching probability p. (**b**), Conductivity of the resulting percolative metallic network above the tiling percolation threshold, following a universal scaling law ($\propto (\langle \phi \rangle - \phi_c)^{1.3}$) [14].

Conductance anisotropy

The electrical properties of the percolated network are modeled as an ensemble average of metallic rotational domains, determined from experimental data. To this end, we consider the polarization dependence of switched nanowires (see also Fig. 3b in the main text) to depend both on the relative absorption as well as on the non-linear switching yield with absorbed fluence. Fig. S8a shows the fluence-dependent switched area fraction $\phi_i(F)$ for 1.2 eV photon energy, where i denotes the nanowire orientation.

Even though the following calculations are performed for 0.8 eV, the threshold characteristic was found to be universal in the investigated energy range. A best fit to the data was found for the phenomenological expression:

$$\phi_i(F) = a_{th} \cdot \arctan((F - f_{th})/w_{th}) + c_{th} \tag{10}$$

where a_{th}, f_{th}, w_{th} and c_{th} denote fit parameters for the threshold amplitude, fluence, width and central value and F is the incident fluence. For 1.2 eV, the nanowire absorption anisotropy is completely diminished by charge carrier delocalization across all domains. For 0.8 eV, however, the absorbed fluence and switched area fraction become orientation-specific. Hence, the polarization dependence of the switched area fraction is fit by an anisotropic absorption:

$$\phi_i(F, \varphi) = a_{th} \cdot \arctan((F \cdot (A_{||}cos(\varphi - \varphi_0)^2 + A_{\perp}sin(\varphi - \varphi_0)^2) - f_{th})/w_{th}) + c_{th} \tag{11}$$

where $A_{||}$, $A_{\perp}$ and φ_0 are fit parameters for the relative absorption and nanowire orientation with respect to the in-plane electric field and φ denotes the incident polarization angle. Fits to the experimental data are depicted in Fig. S8b.

In the simulation of a percolative conduction network, we choose a corresponding F for

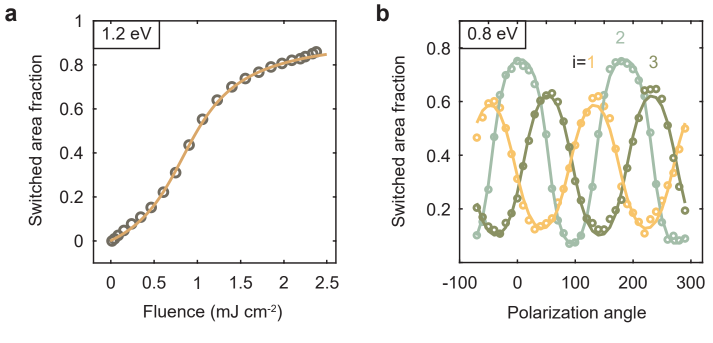

Supplementary Fig. S8: Fits to experimental data. (**a**), Fluence-dependent isotropically switched surface fraction at 1.2 eV incident photon energy (markers) and fit to the experimental data (line). (**b**) Polarization-dependent and domain-resolved switched surface fraction at 0.8 eV incident photon energy (markers) and fit to the experimental data (lines).

every φ so that the mean switched surface area:

$$\langle \phi \rangle = \sum_{i=1}^{3} \phi_i(F_{0.6}, \varphi)/3 \tag{12}$$

reaches a constant value of 0.6, well above the percolation threshold (see Methods: Conductivity in a percolation network). In this way, the composition of the metallic network of rotational domains is weighted by the orientation-resolved relative fraction of switched domains $(\phi_i(F_{0.6}, \varphi)/(3\langle \phi \rangle))$.

Based on the experimental data, we calculate the anisotropic resistivity tensor of the rotational domain texture from the ensemble mean. To this end, we follow established procedures for anisotropic conductive fillers, embedded in an insulating matrix [17, 18].

The intrinsic in-plane resistivity tensor of indium nanowires is:

$$\rho = \begin{pmatrix} \rho_{||} & 0 \\ 0 & \rho_{\perp} \end{pmatrix} \tag{13}$$

where $\rho_{||} = 1/\sigma_{||} = 1.4 \times 10^3\, \Omega/square$ and $\rho_{\perp} = 1/\sigma_{\perp} = 8.3 \times 10^4\, \Omega/square$ are taken from literature [19]. Within the ensemble of rotational domains, the mean resistivity $\langle \rho \rangle$ is expressed in terms of a tensor rotation by the angle of nanowires θ with respect to the above parallel axis [20, 21]:

$$\bar{\rho} = (\rho_{||} + \rho_{\perp})/2 \tag{14}$$

$$\Delta\rho = (\rho_{||} - \rho_{\perp})/2 \tag{15}$$

$$\langle \rho \rangle = \begin{pmatrix} \bar{\rho} + \Delta\rho\langle\cos(2\theta)\rangle & \Delta\rho\langle\sin(2\theta)\rangle \\ \Delta\rho\langle\sin(2\theta)\rangle & \bar{\rho} - \Delta\rho\langle\cos(2\theta)\rangle \end{pmatrix} \tag{16}$$

The angular dependence is calculated with respect to one nanowire orientation ($i = 2$ in Fig. S8b), such that $\theta_1 = -2\pi/3$, $\theta_2 = 0$ and $\theta_3 = 2\pi/3$:

$$\langle\cos(2\theta)\rangle = (\phi_1(F_{0.6}, \varphi)\cos(2\theta_1) + \phi_2(F_{0.6}, \varphi)\cos(2\theta_2) + \phi_3(F_{0.6}, \varphi)\cos(2\theta_3))/3\langle\phi\rangle \tag{17}$$

$$\langle\sin(2\theta)\rangle = (\phi_1(F_{0.6}, \varphi)\sin(2\theta_1) + \phi_2(F_{0.6}, \varphi)\sin(2\theta_2) + \phi_3(F_{0.6}, \varphi)\sin(2\theta_3))/3\langle\phi\rangle \tag{18}$$

Diagonalization of the mean resistivity yields the anisotropy ratio, i.e. the ratio of diagonal components, with respect to the polarization-dependent principal axes (see Extended Data Fig. 4). In the depicted Hall bar geometry, the current $\mathbf{J}$ is constrained in the x-direction ($\mathbf{J} = J_x$). The resulting electric field ratio is thus calculated as:

$$E_y/E_x = \langle\rho\rangle_{xy}/\langle\rho\rangle_{xx} = \Delta\rho\langle\sin(2\theta)\rangle/(\bar{\rho} + \Delta\rho\langle\cos(2\theta)\rangle) \tag{19}$$

Supplementary information references

[1] Wippermann, S. & Schmidt, W. G. Entropy explains metal-insulator transition of the Si(111)-In nanowire array. *Physical Review Letters* **105** (12), 126102 (2010) .

[2] Jeckelmann, E., Sanna, S., Schmidt, W. G., Speiser, E. & Esser, N. Grand canonical Peierls transition in In/Si(111). *Physical Review B* **93** (24), 241407 (2016) .

[3] Böckmann, H., Horstmann, J. G., Razzaq, A. S., Wippermann, S. & Ropers, C. Mode-selective ballistic pathway to a metastable electronic phase. *Structural Dynamics* **9** (4), 045102 (2022) .

[4] Horstmann, J. G. *et al.* Coherent control of a surface structural phase transition. *Nature* **583** (7815), 232–236 (2020) .

[5] Nicholson, C. W. *et al.* Beyond the molecular movie: Dynamics of bands and bonds during a photoinduced phase transition. *Science* **362** (6416), 821–825 (2018) .

[6] Speiser, E., Esser, N., Wippermann, S. & Schmidt, W. G. Surface vibrational Raman modes of In:Si(111) (4×1) and (8×2) nanowires. *Physical Review B* **94** (7), 075417 (2016) .

[7] Peierls, R. E. *Quantum Theory of Solids* (Oxford University Press, 2001).

[8] Yamamoto, S. Optical characterization of ground states of polyacene. *Physical Review B* **78** (23), 235205 (2008) .

[9] Gebhard, F., Born, K., Scheidler, M., Thomas, P. & Koch, S. W. Optical absorption of strongly correlated half-filled Mott-Hubbard chains. *Philosophical Magazine B* **75** (1), 47–65 (1997) .

[10] Song, S. K., Samad, A., Wippermann, S. & Yeom, H. W. Dynamical Metal to Charge-Density-Wave Junctions in an Atomic Wire Array. *Nano Letters* **19** (8), 5769–5773 (2019) .

[11] Shim, H. & Lee, G. True First-Order Surface Phase Transition without Nanoscale Phase Separation. *ACS Nano* **17** (12), 11764–11770 (2023) .

[12] Bollobás, B. & Riordan, O. The critical probability for random Voronoi percolation in the plane is 1/2. *Probability Theory and Related Fields* **136** (3), 417–468 (2006) .

[13] Becker, A. M. & Ziff, R. M. Percolation thresholds on two-dimensional Voronoi networks and Delaunay triangulations. *Physical Review E* **80** (4), 041101 (2009) .

[14] Sarikhani, N., Arabshahi, Z. S., Saberi, A. A. & Moshfegh, A. Z. Unified modeling and experimental realization of electrical and thermal percolation in polymer composites. *Applied Physics Reviews* **9** (4), 041403 (2022) .

[15] Leath, P. L. Cluster size and boundary distribution near percolation threshold. *Physical Review B* **14** (11), 5046–5055 (1976) .

[16] Stauffer, D. & Aharony, A. *Introduction To Percolation Theory* 0 edn (Taylor & Francis, 2018).

[17] Kumar, A. & Kulkarni, G. U. Evaluating conducting network based transparent electrodes from geometrical considerations. *Journal of Applied Physics* **119** (1), 015102 (2016) .

[18] Tarasevich, Y. Y., Vodolazskaya, I. V. & Eserkepov, A. V. Effective electrical conductivity of random resistor networks generated using a Poisson–Voronoi tessellation. *Applied Physics Letters* **123** (26), 263501 (2023) .

[19] Kanagawa, T. *et al.* Anisotropy in Conductance of a Quasi-One-Dimensional Metallic Surface State Measured by a Square Micro-Four-Point Probe Method. *Physical Review Letters* **91** (3), 036805 (2003) .

[20] Frenkel, D. & Eppenga, R. Evidence for algebraic orientational order in a two-dimensional hard-core nematic. *Physical Review A* **31** (3), 1776–1787 (1985) .

[21] Wu, J. *et al.* Angle-Resolved Transport Measurements Reveal Electronic Nematicity in Cuprate Superconductors. *Journal of Superconductivity and Novel Magnetism* **33** (1), 87–92 (2020) .
